# Compatibilization of Polyamide 6/Cyclic Olefinic Copolymer Blends for the Development of Multifunctional Thermoplastic Composites with Self-Healing Capability

**DOI:** 10.3390/ma17081880

**Published:** 2024-04-18

**Authors:** Davide Perin, Andrea Dorigato, Alessandro Pegoretti

**Affiliations:** Department of Industrial Engineering and INSTM Research Unit, University of Trento, Via Sommarive 9, 38123 Trento, Italy; andrea.dorigato@unitn.it (A.D.); alessandro.pegoretti@unitn.it (A.P.)

**Keywords:** polyamide, cyclic olefinic copolymer, blend, compatibilizers, fracture toughness, self-healing

## Abstract

This study investigated the self-healing properties of PA6/COC blends, in particular, the impact of three compatibilizers on the rheological, microstructural, and thermomechanical properties. Dynamic rheological analysis revealed that ethylene glycidyl methacrylate (E-GMA) played a crucial role in reducing interfacial tension and promoting PA6 chain entanglement with COC domains. Mechanical tests showed that poly(ethylene)-graft-maleic anhydride (PE-g-MAH) and polyolefin elastomer-graft-maleic anhydride (POE-g-MAH) compatibilizers enhanced elongation at break, while E-GMA had a milder effect. A thermal healing process at 140 °C for 1 h was carried out on specimens broken in fracture toughness tests, performed under quasi-static and impact conditions, and healing efficiency (HE) was evaluated as the ratio of critical stress intensity factors of healed and virgin samples. All the compatibilizers increased HE, especially E-GMA, achieving 28.5% and 68% in quasi-static and impact conditions, respectively. SEM images of specimens tested in quasi-static conditions showed that all the compatibilizers induced PA6 plasticization and crack corrugation, thus hindering COC flow in the crack zone. Conversely, under impact conditions, E-GMA led to the formation of brittle fractures with planar surfaces, promoting COC flow and thus higher HE values. This study demonstrated that compatibilizers, loading mode, and fracture surface morphologies strongly influenced self-healing performance.

## 1. Introduction

Nowadays, fiber-reinforced polymer composites (FRPCs) have become a popular choice for several applicative fields, including the automotive, aerospace, and renewable energy sectors [1]. Owing to their peculiar characteristics, such as high mechanical properties, processability, lightweight, and corrosion resistance, FRPC use has significantly increased in recent years. According to Reux et al. [2], the main industries using composite materials, in terms of volume, are transportation (28%), building (20%), electric and electronic (16%), pipelines, and tanks (15%). The worldwide composite market is expected to increase from USD 228 billion in 2019 to USD 375 billion by 2026, with a compound annual growth rate (CAGR) of 7.3%. Given the current production rate of composite materials and the estimated future market expansion, an inevitable increase in the amount of composite waste generated can be expected [3]. An important challenge for future FRPCs will be how to handle and recycle them to comply with environmental limits and recently introduced legislation [4]. Consequently, it is crucial to explore sustainable solutions for handling and reusing composite waste to minimize the environmental impact and optimize resource utilization [5,6,7].

One of the most innovative approaches for reducing waste accumulation and prolonging the lifetime and thus the sustainability of composite materials is based on the concept of self-healing [8], i.e., whether damaged self-healing materials have the ability to restore (partially or totally) the original materials’ properties [9]. In their service life, FRPC components are subjected to different types of damage, such as macrodamages from impacts [10] or microdamages from fatigue [11]. The concept of repairing, and thus eliminating, the damage through a self-healing mechanism paves the way for an enhanced lifetime, thus increasing the environmental sustainability of polymer composites. Self-healing materials are generally classified into two broad categories: intrinsic and extrinsic. Intrinsic self-healing materials are based on the concept of repair through physical [12] or chemical [13] interactions at the molecular level. On the other hand, extrinsic self-healing materials utilize pre-embedded healing agents contained in capsules [14,15] or vascular networks [16] that can be released when the crack propagates within the material. When the crack is filled, an irreversible polymerization of the healing agent occurs.

Intrinsic self-healing materials possess a significative advantage over extrinsic ones since the healing process can be potentially repeated multiple times. For this reason, many researchers have recently focused their attention on intrinsic self-healing polymers. The most studied intrinsic self-healing systems, based on chemical processes, rely on the development of covalent [17], free-radical [18], or supramolecular [19,20] dynamic bonds within the material. On the other hand, the most studied physical intrinsic self-healing systems are based on interchain diffusion [21], shape memory effects [22,23], or the generation of a phase-separated morphology [24]. One of the most investigated approaches in the literature is based on the exploitation of miscible [25] or immiscible [26] polymer blends. The healing principle of miscible/immiscible blends relies both on physical and chemical phenomena, and the healing process is performed in five different stages: surface rearrangement, surface approach, wetting, diffusion, and randomization [27]. Miscible/immiscible polymer blends were exploited by Hayes et al. [28] for producing glass fiber-reinforced composites, in which an impact damage area recovery of 30% after a thermal healing process was obtained. The use of immiscible thermoplastic/thermosetting blends for producing intrinsic self-healing materials has been extensively studied in the literature. Meure et al. [29] reported the use of a particulate polyethylene-co-methacrylic acid (EMAA) thermoplastic phase embedded in an epoxy matrix, obtaining up to an 85% recovery of the critical stress intensity factor (K_IC_) after a healing process at 150 °C for 30 min. Mahmood et al. [30] blended different amounts of cyclic olefinic copolymer (COC) in epoxy resin in order to obtain a self-healing matrix. Through the introduction of 40 wt% of COC, they obtained, after thermal treatment of 1 h at 190 °C, a healing efficiency of nearly 100%. Given this result, Dorigato et al. [31] optimized the healing capability of this system by detecting the most suitable conditions in terms of COC content, healing temperature, and pressure. They were able to maintain an excellent healing performance (HE of nearly 80%), repairing the materials at 145 °C for 1 h, and applying a pressure of just 0.5 MPa.

The vast majority of research activities on this topic focus on the development of thermosetting self-healing composites, while the investigation of thermoplastic self-healing composites is still quite limited. The main advantages of thermoplastic composites over thermosetting ones are their recyclability and easier manufacturing. If these positive aspects are coupled with self-healing properties, then thermoplastic composites would potentially replace traditional thermosetting composites in a vast variety of applications [32]. In a previous work of our group, polycaprolactone (PCL) was blended with polyamide 6 (PA6) to obtain novel thermoplastic self-healing polymer blends for structural composites, exploiting the immiscibility between PCL and PA6 [33]. The introduction of 30 wt% of PCL enabled to obtain limited healing efficiency values (up to 6%) in quasi-static mode, while an interesting repair capability (53%) was detected under impact conditions. In a recent paper of our group, COC was blended with PA6 to produce a multifunctional thermoplastic matrix for composites with self-healing properties [34]. Due to the immiscibility of the prepared blends, the introduction of 30 wt% of COC led to a healing efficiency of 11% in quasi-static mode and 35% in impact mode. In this work, the authors explained that the limited HE values obtained were related to the lack of interfacial adhesion between the matrix and the healing agent.

Based on these considerations, the present work aims to improve the interfacial adhesion between COC and PA6 without hindering the flow of the softened healing agent (i.e., COC) during the repair process. With this aim, three different compatibilizers were taken into consideration: poly(ethylene)-graft-maleic anhydride (PE-g-MAH), polyolefin elastomer-graft-maleic anhydride (POE-g-MAH), and ethylene-glycidyl methacrylate (E-GMA). The blends, prepared through melt compounding and hot pressing and keeping a constant compatibilizer amount (5 wt%), were subjected to a comprehensive rheological, microstructural, and thermomechanical characterization in order to compare the effect of the different compatibilizers on the healing behavior of the prepared matrices.

## 2. Materials and Methods

### 2.1. Materials

**Figure 1:**
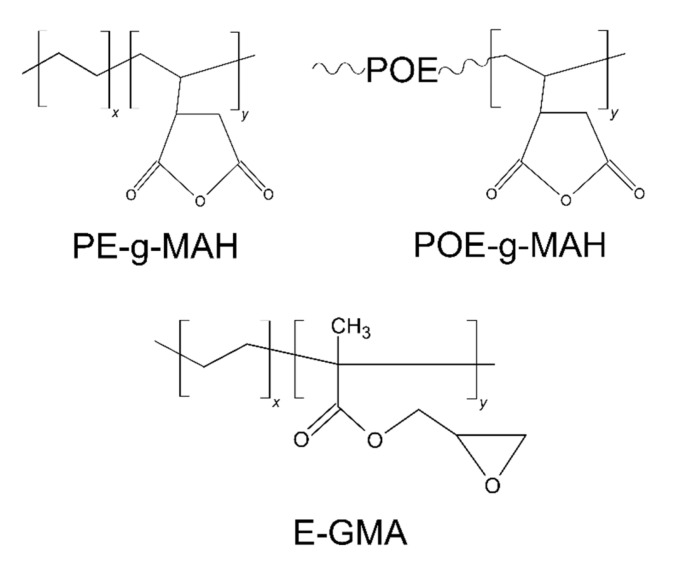
Schematization of the chemical structure of the selected compatibilizers.

Two different thermoplastic materials were selected for the production of the self-healing blends: polyamide 6 (PA6) and cyclic olefinic copolymer (COC). PA6 was a Radilon S 24E 100 NAT, kindly provided by Radici Group SpA (Gandino, Italy) in pellet form, with a density of 1.14 g/cm^3^ and a melting temperature of 220 °C. The healing agent was a commercial grade COC known as Topas COC 9506F-500, produced by TOPAS Advanced Polymers GmbH (Raunheim, Germany) and delivered in pellet form with a density of 1.01 g/cm^3^. The glass transition temperature (T_g_) of the COC was 65 °C, and it had a norbornene content of 61 wt%. To improve the interaction between the constituents of the blend, the following three different compatibilizers were considered: poly(ethylene)-graft-maleic anhydride (PE-g-MAH), polyolefin elastomer-graft-maleic anhydride (POE-g-MAH), and poly(ethylene-co-glycidyl methacrylate) (E-GMA). PE-g-MAH and POE-g-MAH were kindly provided by Auserpolimeri Srl (Milan, Italy) in granule form and were commercially distributed under the names CO/PA UL and CO/PA 160H, respectively. PE-g-MAH had a density of 0.88 g/cm^3^ and a melt flow index (MFI) of 32 g/10 min at 230 °C with an applied mass of 10 kg. POE-g-MAH had a density of 0.92 g/cm^3^ and an MFI of 6 g/min at 230 °C with an applied mass of 10 kg. The grafting level information was protected by the company. The third compatibilizer, E-GMA, was procured from Merck KGaA (Darmstadt, Germany) in granule form. It was characterized by an MFI at 190 °C of 5 g/10 min and a glycidyl methacrylate content ranging between 6.5 and 9.0 wt%. This compatibilizer was chosen due to the potential for a chemical reaction between PA6 and the compatibilizer, with only a partial physical interaction with the COC. By enhancing interfacial adhesion without impairing the flow of the healing agent in the crack zone at the healing temperature, a better repair could be obtained. Figure 1 depicts the chemical structures of the selected compatibilizers.

### 2.2. Sample Preparation

To prevent hydrolytic degradation during the melt mixing process, PA6 granules underwent drying at 80 °C for 12 h in a vacuum oven, while COC pellets and compatibilizers were dried at 50 °C for 12 h in a ventilated oven. Initially, both PA6 and COC granules were melt-compounded with a constant PA6/COC weight ratio of 70/30 in a Haake PolyLab system (Karlsruhe, Germany) consisting of a Rheomix 600 internal mixer, equipped with counter-rotating rotors operating at 60 rpm at a temperature of 230 °C for 1 min. Subsequently, 5 wt% of compatibilizer was added, resulting in a total processing time of 6 min. The PA6/COC ratio was selected based on a previous study of our group [34], while the compatibilizer concentration was determined according to the literature data and the recommendations of the companies providing the compatibilizers. The resulting blends were then compression-molded in a Carver hot plate press at 235 °C for 8 min, under an applied pressure of 3.4 MPa. By using this method, square sheets with two different thicknesses (120 × 120 × 2 mm^3^ and 100 × 100 × 5 mm^3^) were prepared. The uncompatibilized blend was referred to as PA6COC, while the compatibilized blends were labeled as 5 wt% followed by the used compatibilizer. Table 1 lists all the prepared samples and the corresponding weight fraction of each constituent.

### 2.3. Experimental Techniques

#### 2.3.1. Rheological Properties

Rheological measurements were carried out using an HR-2 Discovery Hybrid Rheometer (TA Instruments, New Castle, DE, USA), which operated in parallel plate configuration (plate diameter = 25 mm), while the loading gap was set at 1.8 mm. This instrument was employed to conduct frequency sweep tests at 230 °C in air, within a frequency range of 0.05 to 600 rad/s, while a strain amplitude of 1% was utilized to maintain the measurements within the linear viscoelastic region. These tests enabled the determination of the trends of storage modulus (G’), loss modulus (G”), and complex viscosity (η*) as a function of frequency. A minimum of three specimens were tested for each composition.

#### 2.3.2. Microstructural and Chemical Properties

Field emission scanning electron microscopy (FESEM) images of the cryofractured surfaces of both virgin and healed samples were obtained through a Zeiss (Oberkochen, Germany) Supra 40 microscope operating at an acceleration voltage of 2.5 kV. A platinum/palladium (80:20) conductive coating was sputtered on the specimens prior to observation to ensure good electrical conductivity. Fourier transform infrared spectroscopy (FT-IR) was carried out in attenuated total reflectance (ATR) mode using a Perkin-Elmer Spectrum One instrument (Perkin Elmer GmbH, Waltham, MA, USA), equipped with a ZnSe crystal, in a wavenumber range of 650–4000 cm^−1^. To enhance the signal-to-noise ratio, twenty scans were collected for each spectrum with a resolution of 4 cm^−1^.

#### 2.3.3. Thermal Properties

Differential scanning calorimetry (DSC) analyses were carried out using a Mettler DSC30 apparatus under a nitrogen flow of 100 mL/min, encompassing a temperature range between −20 and 250 °C, with a heating/cooling rate of 10 °C/min. The thermograms obtained were utilized to detect the thermal transitions of the blend constituents, such as the glass transition temperature (T_g_) of the COC, the melting temperature and enthalpy (T_m_, ΔH_m_) of PA6, and the crystallization temperature and enthalpy (T_c_, ΔH_c_) of PA6. Equation (1) shows the relation utilized to assess the degree of crystallinity (χ) of the PA6 phase in the blends:

(1) $$\chi =\frac{\Delta H_{m}-\Delta H_{cc}}{\Delta H_{m}^{*}\;\cdot \;\omega_{PA6}}\;\cdot 100$$

where ΔH_cc_ is the enthalpy of cold crystallization of the PA6, $\Delta H_{m}^{*}$ is the enthalpy of melting of fully crystalline PA6, taken equal to 230 J/g [35], and ω_PA6_ is the weight fraction of PA6 in the blend.

#### 2.3.4. Mechanical Properties

An Instron^®^ 5969 tensile testing machine (manufactured by ITW Test & Measurement and Equipment, Norwood, MA, USA) was utilized to perform quasi-static tensile tests at ambient temperature. The testing machine was equipped with a 1 kN load cell and the tests were performed according to ISO 527 standard [36] using 1BA specimens, having a gauge length of 30 mm. The crosshead speed was set at 10 mm/min for the tests conducted at break. A minimum of ten specimens were tested for each composition. In this way, the maximum stress (σ_max_) and strain at break (ε_b_) were determined. To calculate the elastic modulus (E), additional tensile tests were conducted using the same machine, but an Instron^®^ 2620-601 extensometer, having a gauge length of 12.5 mm, was applied to the specimens, and a lower testing speed (0.25 mm/min) was utilized. The elastic modulus was determined in accordance with ISO 527 standard, considering the stress levels corresponding to strain values of 0.05% and 0.25%. A minimum of five specimens were tested for each formulation.

The assessment of the fracture toughness of the compatibilized blends was carried out using single edge notched bending (SENB) specimens, which measured 44 × 10 × 5 mm^3^, and featured a notch with a depth of 5 mm and a span length of 40 mm. The tests were conducted in accordance with ASTM D5045 standard [37] using an Instron^®^ 5969 electromechanical testing machine. The three-point bending tests on the notched specimens were performed at a crosshead speed of 10 mm/min, with at least 12 specimens tested for each composition. In addition, impact mode tests were carried out using a CEAST impact machine equipped with a 0.5 kg hammer, an initial angle of 60°, and an impact speed of 1.5 m/s. A minimum of 12 specimens were tested for each formulation. From the load–displacement curves, the maximum load sustained by the samples (P_max_) was determined, allowing for the calculation of the critical stress intensity factor (K_IC_) in both quasi-static and impact conditions, as specified in Equation (2):

(2) $$K_{IC}=\frac{P_{MAX}}{tw^{1/2}}\;\cdot f\left(x\right)$$

where t is the thickness of the sample, w is the width of the samples, and f(x) is a calibration factor defined by ASTM D5045 standard, being x = a/w the ratio between the notch depth and the width of the specimens. From the integration of the load–displacement curves and the evaluation of the system compliance, the critical strain energy release rate (G_IC_) values in quasi-static mode were calculated, according to the expression reported in Equation (3):

(3) $$G_{IC}=\frac{\Delta U}{BW\varphi }\;$$

where ΔU is the difference between the total energy absorbed by the specimens and the energy absorbed in the indentation tests, while φ is an energy calibration factor, whose expression is reported in ASTM D5045 standard.

#### 2.3.5. Evaluation of the Healing Efficiency

In the literature, the effectiveness of the healing process is commonly described in terms of healing efficiency (η), which is defined in different ways by the authors [38]. Typically, η is expressed as a relative percentage based on mechanical properties, such as strength, stiffness, or toughness. In this study, a thermal mending process was carried out on the prepared blends. The specimens used for fracture toughness tests performed both under quasi-static and impact conditions, were repaired using a lab-made device (detailed in [33,34]), by placing them in an iron vice with an applied pressure of 0.5 MPa and heating them in an oven at 140 °C for 60 min. These healing parameters were chosen after preliminary lab trials [34]. The specimens that underwent the thermal mending process were tested again both in quasi-static and impact mode, and the fracture toughness of the healed specimens (K_IC,Healed_) was obtained. Thus, the healing efficiency (η_KIC_) was evaluated using the expression reported in Equation (4):

(4) $$\eta_{KIC}=\frac{K_{IC\_Healed}}{K_{IC\_Virgin}}\;\cdot 100$$

where K_IC_virgin_ is the critical stress intensity factor of the virgin specimens and K_IC_Healed_ is the critical stress intensity factor of the healed specimens.

## 3. Results and Discussions

### 3.1. Rheological Properties

**Figure 2:**
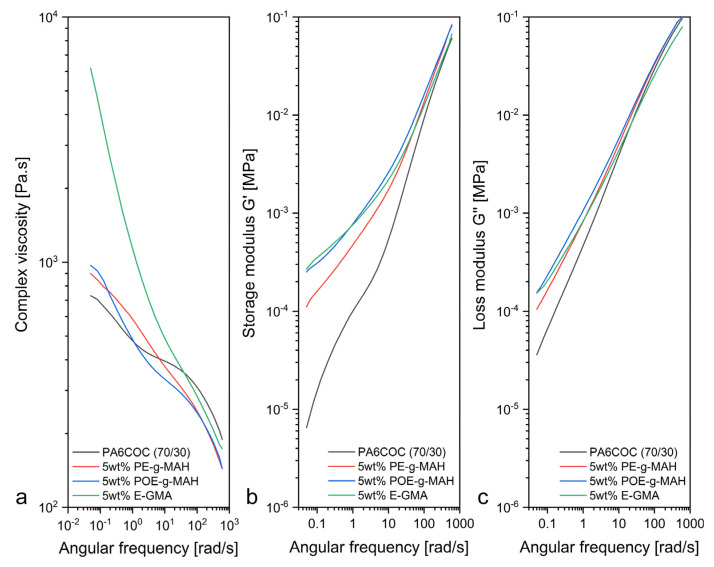
Dynamic rheological behavior of the prepared PA6/COC blends. Trends of (**a**) complex viscosity, (**b**) storage modulus, and (**c**) loss modulus as a function of the angular frequency at 230 °C.

A comprehensive analysis was carried out to investigate the rheological properties of the produced blends in order to evaluate their processability and their miscibility upon compatibilization. Figure 2a–c report the results obtained from dynamic rheological measurements on the prepared blends in terms of dynamic moduli (G’, G”) and complex viscosity (η*) at 230 °C. The introduction of 5 wt% of compatibilizer into the PA6/COC system increases the storage modulus over the entire range of frequency, and this enhancement is more appreciable in the low-frequency region. The low-frequency G’ values of the compatibilized blends (5 wt% PE-g-MAH, 5 wt% POE-g-MAH, and 5 wt% E-GMA) are systematically higher in comparison with the uncompatibilized system. The increase in G’ upon compatibilization has already been observed by several authors in other polymer blends. Krache et al. [39] investigated the effect of polystyrene–poly(ethylene butylene)–polystyrene copolymer (SEBS) and SEBS-grafted maleic anhydride (SEBS-g-MAH) on the morphology of binary and ternary blends of polyethylene (PE), polypropylene (PP), and polyamide 6,6 (PA6,6). In their work, they noted a remarkable increase in G′, especially at low frequencies, due to the presence of the compatibilizer. Basseri et al. [40] studied a PP/poly(styrene-co-acrylonitrile) (SAN) blend containing poly(styrene-b-butadiene-b-styrene) (SBS). By performing dynamic rheology measurements, they noticed an appreciable increase in G’ at low frequencies when 20 wt% of SBS was introduced. In the present paper, the addition of 5 wt% of POE-g-MAH and 5 wt% E-GMA determines a significant G’ increase in the low-frequency range, while the introduction of 5 wt% PE-g-MAH only slightly increases G’. Furthermore, the storage modulus curve of all the compatibilized blends reports a non-terminal trend at low frequencies. Thus, it is appreciable that the G’ of the blend compatibilized with E-GMA and POE-g-MAH is greater than that observed in the other compatibilized systems. This G’ increase suggests a significant hindrance to the mobility and the molecular relaxation of the COC phase [41]. Referring to Figure 2a, the incorporation of the compatibilizers in the PA6/COC blend results in a complex viscosity increase over the entire range of frequency, especially in the low-frequency interval. This η* increment is the result of improved interfacial interaction between the PA6 matrix and COC domains due to the presence of the compatibilizers. However, the incorporation of PE-g-MAH and POE-g-MAH yields merely a slight increase in the complex viscosity, while the addition of E-GMA results in a noticeable η* enhancement at low frequencies. In order to explain this trend, it is possible to hypothesize that, during the melt mixing phase, the epoxide rings of E-GMA chemically react with amine end groups and/or amidic bonds of the PA6 chains, thus leading to the formation of a PA-g-EGMA copolymer located in the interfacial region [42], producing thus a strong enhancement of the adhesion and thus a better chain entanglement between the PA6 and COC phases.

### 3.2. Microstructural and Chemical Properties

**Figure 3:**
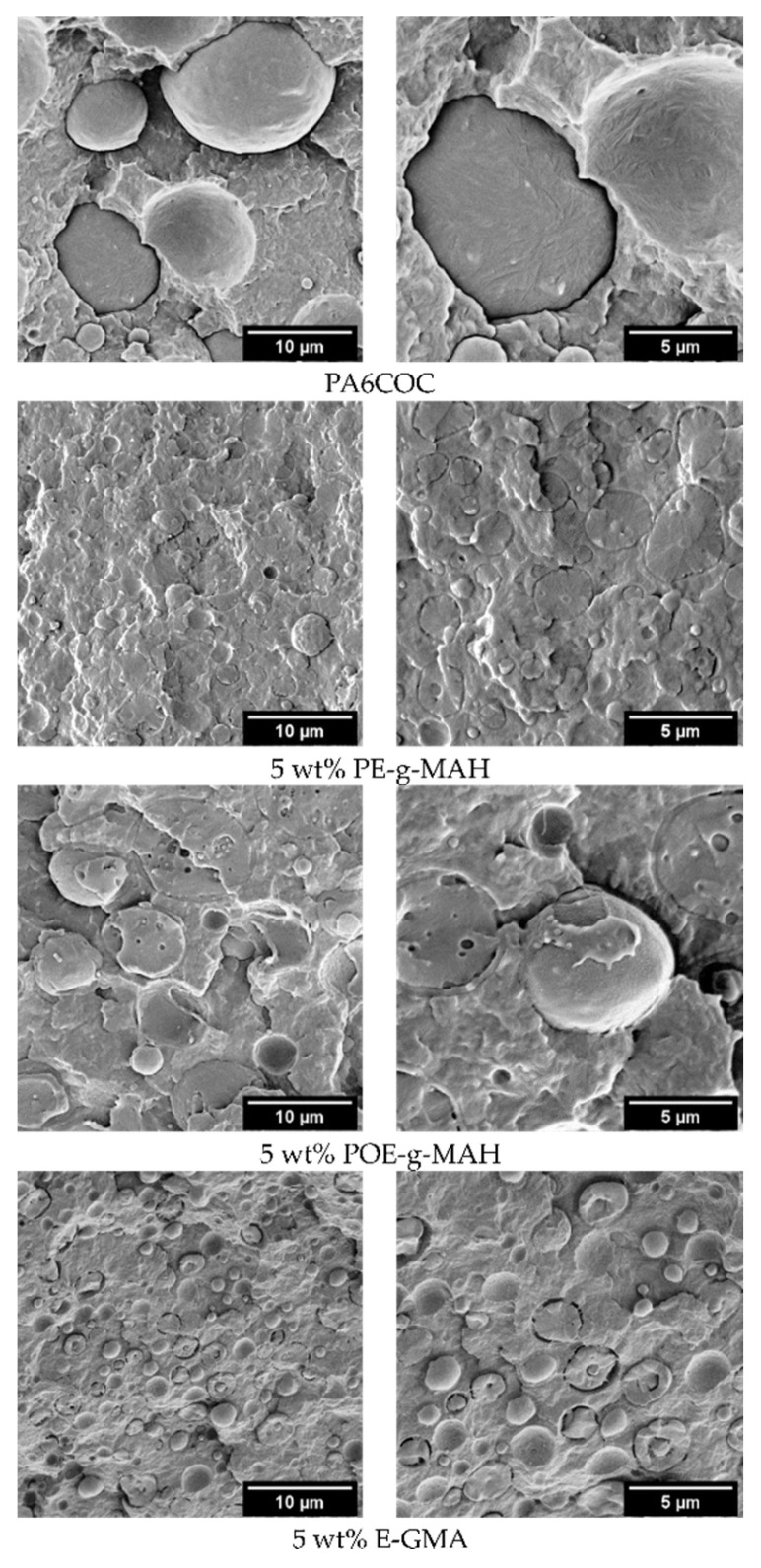
FESEM micrographs of the cryofractured surfaces of the prepared blends at two different magnification levels.

From the examination of the cross-section of the prepared blends through light microscopy (not reported for the sake of brevity, see Figure S1 in the Supplementary Materials), it can be observed that the prepared blends are characterized by the typical morphology of immiscible blends, with domains of COC homogenously distributed within the PA6 matrix. The average diameter of COC domains in the uncompatibilized blend is 23.4 ± 5.1 µm, while the COC domain size strongly decreases upon the addition of the compatibilizers. Through the introduction of PE-g-MAH, POE-g-MAH, and E-GMA, the average COC domain diameter decreases to 4.0 ± 0.8 µm, 6.8 ± 1.3 µm, and 2.9 ± 0.5 µm, respectively. Even if from the obtained micrographs it is possible to infer that the compatibilized blends are still immiscible, a strong refinement of the morphology can be obtained thanks to the addition of the compatibilizers. The phase morphology of the prepared blends is also investigated through FESEM, and Figure 3 reports the micrographs of the cryofractured surfaces of the prepared samples. As expected, the morphology of all the blends is characterized by an evident phase separation [43]. COC domains in the uncompatibilized blend are almost completely detached from the PA6 matrix, with a very limited interfacial adhesion between the two constituents. On the other hand, by introducing the compatibilizers, different types of interphase can be observed. By looking at the effect of PE-g-MAH, it is evident that the interphase becomes so strong that, during the preparation of the cryofractured surfaces, the crack propagates within the COC domains instead of producing its detachment from the PA6 matrix. This provides compelling proof of the compatibilization effectiveness of PE-g-MAH, also confirmed by other works on PA6/polyethylene (PE) blends [44,45,46,47]. By introducing POE-g-MAH, a weaker interphase is formed around the COC domains, and the crack produces an evident interfacial debonding. By observing the effect of E-GMA, it is possible to notice that the COC domains are very small, characterized by an irregular shape and partially bonded to the PA6 matrix. It is well known that E-GMA and PA6 have good chemical compatibility, while between COC and E-GMA there is only the presence of chain entanglement [48]. It is important to underline that the obtained results are consistent with those obtained from the dynamic rheological measurements (see Figure 2a–c).

**Figure 4:**
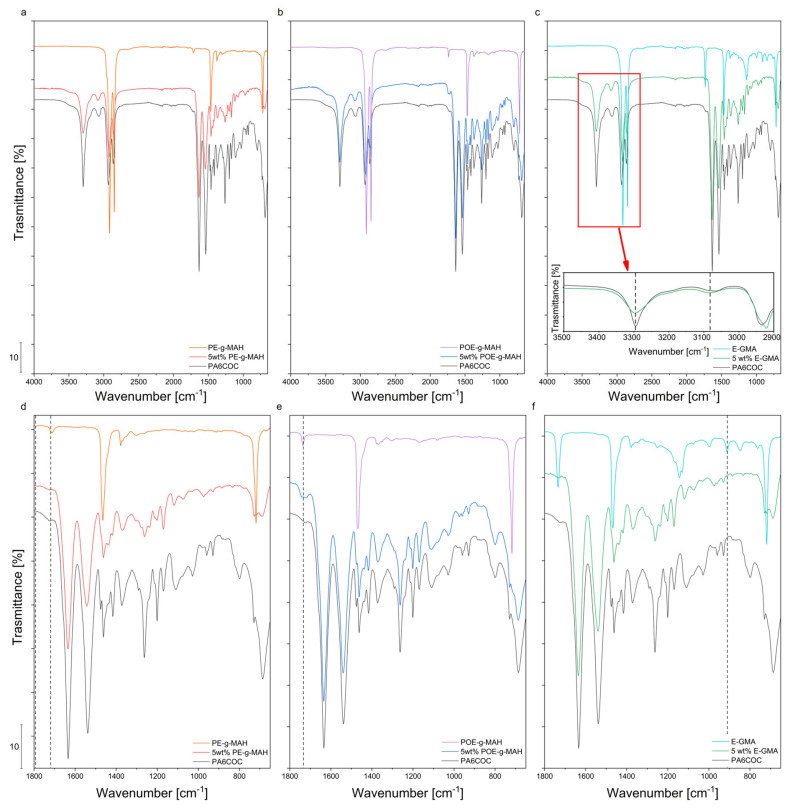
ATR-FTIR spectra of the PA6/COC blends compatibilized with (**a**) PE-g-MAH, (**b**) POE-g-MAH, and (**c**) E-GMA; (**d**–**f**) detail of the same spectra in the 650–1800 cm^−1^ wavenumber range.

Figure 4a–f report the FTIR spectra of the prepared blends. PA6 phase is characterized by several characteristic peaks showing major absorption bands at 3295, 3075, 2925, and 2866 cm^−1^. In particular, the two broad absorption peaks at 3295 and 3075 cm^−1^ can be correlated to the N–H hydrogen bond stretching vibration and hydroxyl bonds, respectively [49]. The signals at 2925 cm^−1^ and at 2866 cm^−1^ correspond to the asymmetric and symmetric C-H stretching vibrations, respectively [50]. The peak located at 1634 cm^−1^ can be assigned to the carbonyl stretching vibration characteristic of amide I band, while the peak at 1538 cm^−1^ can be correlated with N–H bending and C–N stretching of amide II band [51]. The presence of the COC phase is highlighted by the characteristic bands of the ethylene–norbornene conjugation (1639 cm^−1^), the ring deformation of norbornene (1596 cm^−1^), and the C–H vibrations of the methylene group (1459 cm^−1^). However, the signals of COC are overlapped by the much stronger signals associated with the PA6 constituent. Concerning the effect of the compatibilizers, the spectrum of PE-g-MAH (see Figure 4a,d) reports the characteristic peaks of cyclic anhydride groups in the 1815–1710 cm^−1^ region, but they are particularly weak given the low amount of maleic anhydride present in the material. The two most important peaks are located at 1791 and 1713 cm^−1^ and can be respectively correlated to asymmetric and symmetric stretching vibrations of maleic anhydride and carboxylate groups of maleic acid [52,53]. The peaks at 1465 and 710 cm^−1^ are associated with the bending and rocking vibrations of -CH_2_ aliphatic groups in PE-g-MAH [54]. In the 5 wt% PE-g-MAH blend, anhydride-related peaks vanish/shift as a result of compatibilization, which should exhibit peaks at 1360 and 715 cm^−1^ due to cyclic imide C-N bonds [46]. These peaks are overlapped by the characteristic signals of other chemical bonds present in the blend and therefore cannot be detected. Only a small signal near 715 cm^−1^ can be noticed, but the signal is overlapped by other peaks. On the other hand, compatibilization is considered to occur through the chemical bond of the anhydride group on the PE-g-MAH chain with the PA6 end groups. This has been evidenced by the rheological measurements and also supported by SEM observations. The effect of POE-g-MAH is similar to the one of PE-g-MAH, and the characteristic peaks related to POE-g-MAH can be observed in Figure 4b,e. The peaks corresponding to POE-g-MAH seem to disappear in the compatibilized blend. The reaction between the amine moieties of PA6 and the maleic anhydride groups of POE-g-MAH generates C-N bonds, and this signal overlaps the peaks characteristic of PA6. The effect of the third compatibilizer, i.e., E-GMA, is slightly different from that detected in the other two. The characteristic peak associated with the C=O stretch of the ester group can be appreciated at 1730 cm^−1^, while there is a peak located at 910 cm^−1^, corresponding to the glycidyl epoxy group [55]. The magnified spectrum inserted in Figure 4f highlights the variation of the peak intensity in the wavelength range 3600–3200 cm^−1^ in the compatibilized blend. As reported in the literature, at 3300 cm^−1^, it is possible to appreciate the peak associated with N–H stretching vibration, while, at 3080 cm^−1^, the signal corresponding to the hydroxyl group can be detected [56]. Upon the introduction of E-GMA, the intensity of the peak located at 3080 cm^−1^ tends to increase, whereas the peak at 3300 cm^−1^ tends to decrease. This experimental evidence seems to suggest that, thanks to the chemical reaction that occurs during the compatibilization with E-GMA, part of the N–H groups of PA6 tend to form hydrogen bonds with C=O groups of the E-GMA compatibilizer [57].

### 3.3. Thermal Properties

**Figure 5:**
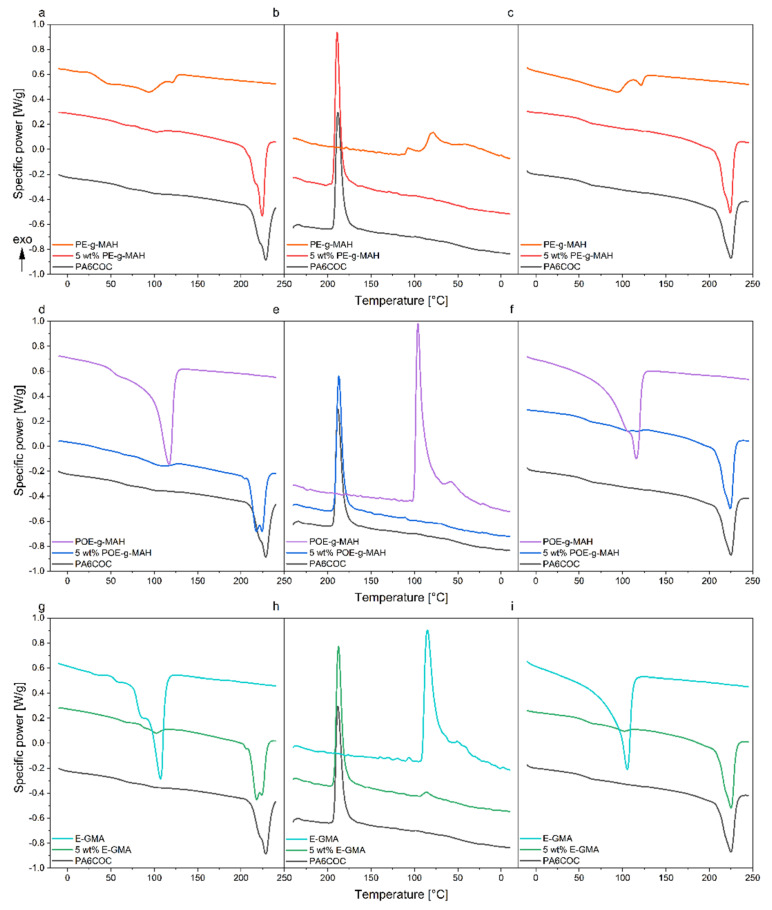
DSC thermograms of the prepared blends and of the relative compatibilizers. (**a**) First heating scan, (**b**) cooling scan, and (**c**) second heating scan of 5 wt% PE-g-MAH; (**d**) first heating scan, (**e**) cooling scan, and (**f**) second heating scan of 5 wt% POE-g-MAH; (**g**) first heating scan, (**h**) cooling scan, and (**i**) second heating scan of 5 wt% E-GMA.

Figure 5a–i report the DSC thermograms of the prepared blends and the relative compatibilizers, whereas the most important results are listed in Table 2. As reported in a previous work of our group [34], the incorporation of COC into the PA6 matrix partially inhibits the formation of stable crystallites (α-phase characterized by a melting temperature T_m2_), promoting the generation of a less stable crystalline phase during cooling (γ-phase with a melting temperature T_m1_). The compatibilized blends exhibit a double melting peak and the temperatures of the first melting peak (T_m1_) are systematically lower in comparison with the PA6COC blend, suggesting the potential formation of less stable crystals within the PA6 matrix [58]. The compatibilization process slightly influences the crystallinity degree of PA6, which passes from 22.7% in the uncompatibilized blend to 27.5% (5 wt% PE-g-MAH), 27.4% (5 wt% POE-g-MAH), and 26.8% (5 wt% E-GMA). The melting peak of the 5 wt% PE-g-MAH sample is characterized by a more intense signal coming from α-phase rather than γ-mesophase. On the other hand, both 5 wt%POE-g-MAH and 5 wt% E-GMA blends are characterized by a strong signal from both α-phase and γ-mesophase. The addition of the three different compatibilizers does not result in an appreciable shift in the T_g_ of COC, confirming the immiscibility of the compatibilized blends. In conclusion, the increase in crystallinity degree of PA6 may be related to an enhanced interaction between PA6 and COC. The noticeable reduction in the dimensions of the COC domains upon compatibilization can probably influence the nucleation process of PA6 crystals.

### 3.4. Mechanical Properties

**Figure 6:**
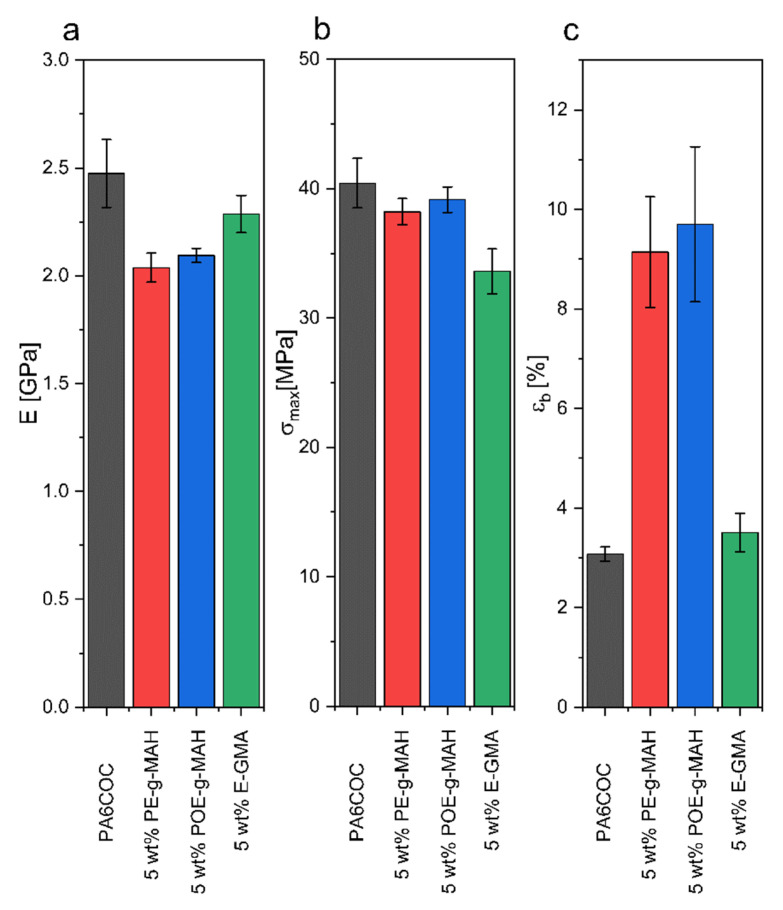
Results of quasi-static tensile tests performed on the prepared blends: (**a**) elastic modulus, (**b**) maximum stress, and (**c**) elongation at break.

Figure 6a–c report the trends of E, σ_max_, and ε_b_ for the prepared blends obtained in quasi-static tensile tests. When PE-g-MAH and POE-g-MAH are introduced, a reduction in the elastic modulus is evident, passing from the value of 2.5 GPa for PA6COC to 2.0 and 2.1 GPa, respectively, while the addition of E-GMA leads to a milder E worsening down to 2.3 GPa. σ_max_ is only slightly reduced upon the introduction of PE-g-MAH and POE-g-MAH. Specifically, PA6COC records a σ_max_ of 40.4 MPa, while PE-g-MAH and POE-g-MAH show σ_max_ values of 38.2 MPa and 39.1 MPa, respectively. A more significant drop can be registered with the incorporation of E-GMA (σ_max_ = 33.6 MPa). Interestingly, the elongation at break experiences a notable increase when PE-g-MAH and POE-g-MAH are added, rising from 3.1% in uncompatibilized blends up to 9.1% and 9.7%, respectively. Instead, the introduction of E-GMA results in a limited ε_b_ enhancement (3.5%). SEM micrographs in Figure 3 reveal a clear lack of interfacial adhesion between the dispersed COC domains and the PA6 matrix in the uncompatibilized blend, and the enhancement of the elongation at break upon the introduction of PE-g-MAH and POE-g-MAH can be primarily attributed to the improvement of the interfacial bonding between PA6 and COC phases. In fact, both the compatibilizers are capable of reacting with the PA6, therefore enhancing the blend miscibility. In contrast, the positive effect on ε_b_ played by E-GMA is more limited. As explained in Section 3.2, E-GMA can react with PA6, whereas only physical entanglements are formed between the COC phase and E-GMA compatibilizer, leading thus to a modest improvement in the elongation at break.

**Figure 7:**
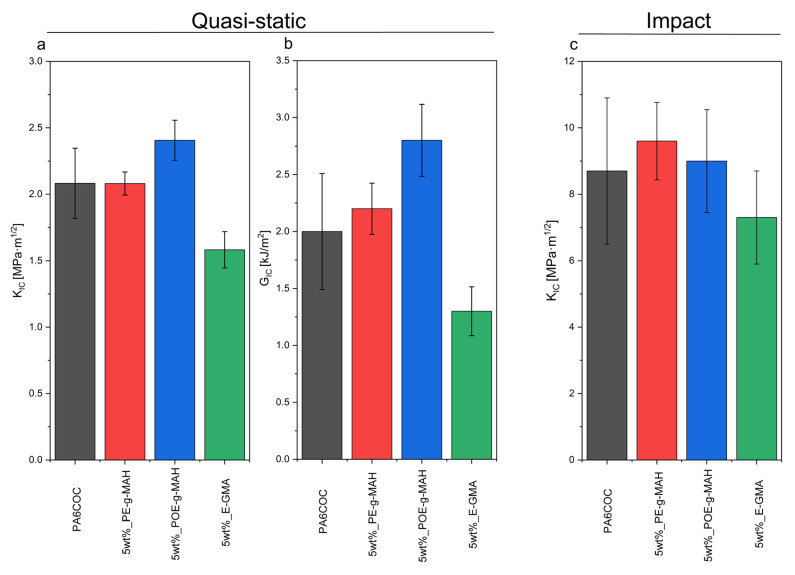
Results of the fracture toughness tests performed on the prepared blends. (**a**) K_IC_ evaluated in quasi-static mode, (**b**) G_IC_ evaluated in quasi-static mode, and (**c**) K_IC_ evaluated in impact mode.

Figure 7a,b shows the trends of K_IC_ and G_IC_ for the prepared blends, tested in quasi-static mode. Notably, the introduction of PE-g-MAH and POE-g-MAH results in an increase in both K_IC_ and G_IC_, while a slight drop can be registered upon the incorporation of E-GMA. The observed enhancement in K_IC_ and G_IC_ values obtained with PE-g-MAH and POE-g-MAH compatibilizers could be attributed to an evident plasticization of the matrix and to interfacial debonding [59]. It could be hypothesized that the detachment of COC particles from the PA6 matrix induces a modification in the stress distribution in the interfacial region, enabling the matrix to yield at relatively modest stress levels and promoting its plastic deformation. In Figure 7c, the K_IC_ values derived from fracture toughness tests conducted under impact mode are shown. It can be appreciated that the K_IC_ values obtained in impact conditions are characterized by the same trend observed in quasi-static mode.

**Figure 8:**
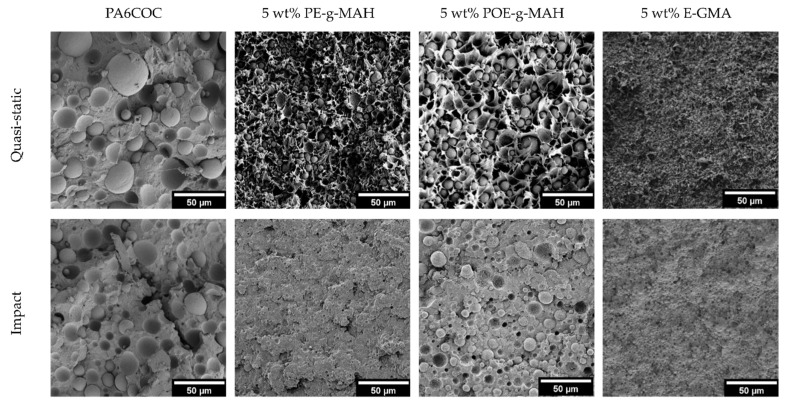
SEM micrographs of the fracture surface of the samples tested both in quasi-static and impact mode.

In order to explain these results, SEM micrographs of the tested SENB specimens are shown in Figure 8. Under quasi-static conditions, the surface of the uncompatibilized specimen is characterized by the presence of voids resulting from the particle debonding process. Conversely, with the introduction of compatibilizers, the PA6 matrix undergoes significant plasticization and is able to absorb a substantial amount of energy to hinder the crack propagation and thus enhance the fracture toughness [60,61]. Conversely, at an elevated testing speed, the toughening mechanism due to the plastic deformation of the matrix does not occur, and the fracture propagates before the PA6/COC interfacial debonding. As a matter of fact, by comparing the fracture surface of uncompatibilized and compatibilized blends, it is possible to notice a similar morphology. The only difference that it is possible to appreciate is the finer distribution of the COC domains in compatibilized blends, especially with the addition of E-GMA compatibilizer. The similar morphology of the fractured specimens could explain the similar K_IC_ values registered under impact conditions.

### 3.5. Evaluation of the Healing Efficiency

**Figure 9:**
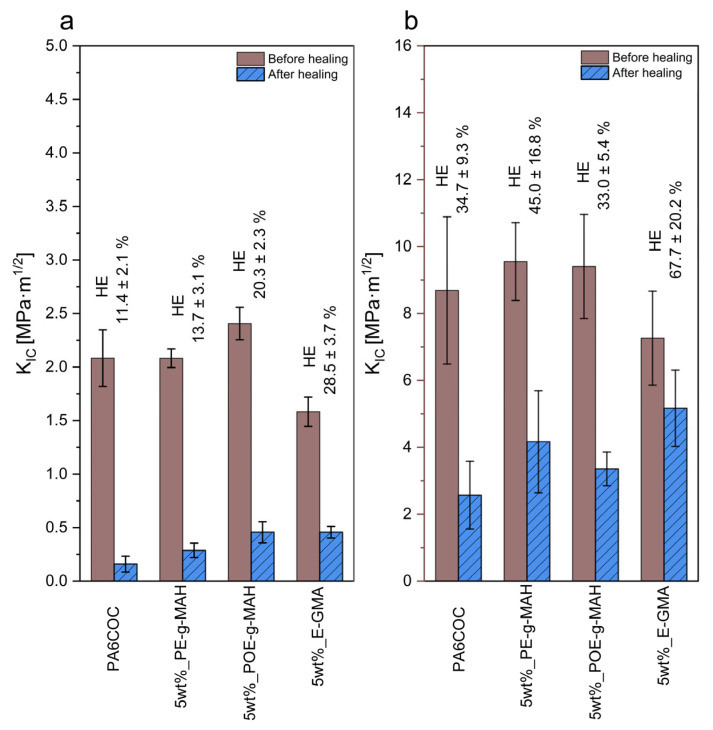
Fracture toughness of the prepared blends before and after the healing process. (**a**) Quasi-static mode and (**b**) impact mode.

Figure 9a,b report the K_IC_ values registered both in quasi-static and impact mode for uncompatibilized and compatibilized blends before and after the healing process. In the case of the uncompatibilized blend, HE observed is 11.4% in quasi-static mode and 34.7% in impact conditions. With the incorporation of compatibilizers, there is a notable improvement in HE values. In quasi-static mode, PE-g-MAH addition leads to a modest HE enhancement up to 13.7%, POE-g-MAH increases HE up to 20.3%, while E-GMA significantly enhances HE up to 28.5%. In impact mode, the influence played by the compatibilizers on HE is even more pronounced. PE-g-MAH addition leads to a mild improvement in HE up to 45.0%, while E-GMA introduction enhances HE up to 67.7%. The evident enhancement in the repair capability of the compatibilized blends can be attributed to an improved interaction between the COC and the PA6 matrix, along with a finer distribution of COC domains. Furthermore, it is noteworthy that the healing efficiency evaluated in impact mode is systematically higher than that observed in quasi-static mode. This phenomenon is closely related to the distinct fracture surface morphology produced by applying different testing speeds.

**Figure 10:**
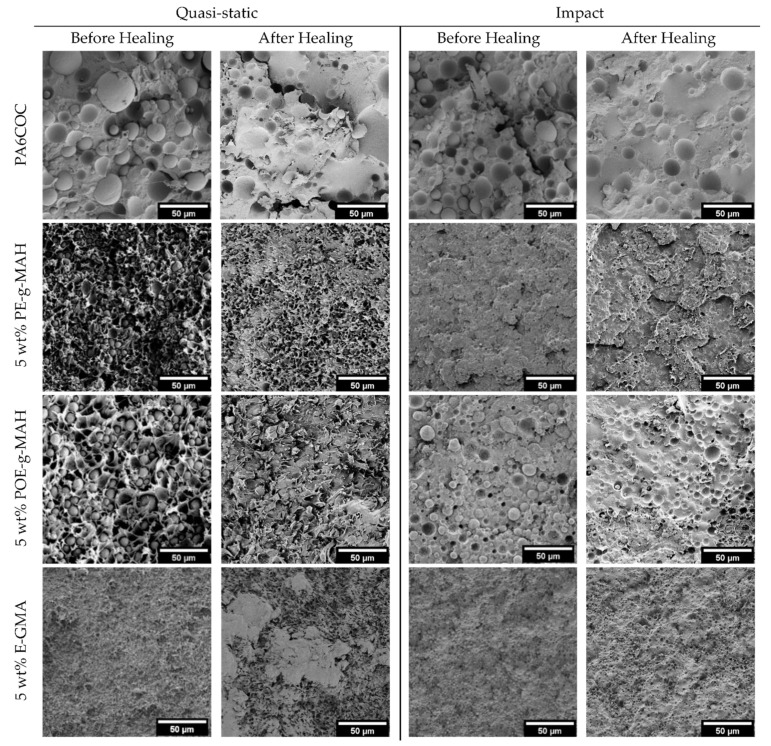
SEM micrographs of the fracture surface of the prepared blends before and after the healing process, tested both in quasi-static and impact conditions.

Figure 10 compares SEM micrographs of the fracture surface of both uncompatibilized and compatibilized blends tested both in quasi-static and impact mode, both before and after the healing procedure. As previously mentioned, the introduction of compatibilizers elicits a substantial plasticization effect on the PA6 matrix in the quasi-static mode, resulting in the generation of a peculiar morphology (see Figure 8). In the subsequent thermal healing process, COC domains tend to soften and flow towards the crack zone. It becomes evident that COC tends to remain segregated in specific zones, without filling the crack. Therefore, the extensive plasticization of the PA6 matrix, with the formation of an irregular crack plane, impedes the optimal filling of the cracks by COC, leading thus to limited HE values. Upon the incorporation of PE-g-MAH and POE-g-MAH compatibilizers, notable enhancements in mechanical properties are observed, due to the PA6 plasticization and the formation of a stronger interphase, but these effects limit the healing capability of the resulting blends. The introduction of E-GMA plays a role in reducing the surface tension of PA6, resulting in smaller COC domains. Furthermore, due to the weaker interfacial interaction produced with respect to the other two compatibilizers, the COC domains can flow more efficiently within the crack zone. The situation changes dramatically in impact conditions. With the surface smooth and more regular than that observed in quasi-static mode (see Figure 8), the COC domains are capable of efficiently filling the crack plane. The micrographs yield evidence of a thin and uniform COC film covering a substantial portion of the crack surface. Furthermore, the improved interphase between COC domains and the PA6 matrix contributes significantly to further increasing HE values.

A pivotal turning point in advancing the comprehension of the self-healing performance of the materials lies in fractography analysis. The sole reliance on healing efficiency values proves inadequate, as the morphology of the crack plane exerts a profound influence on the efficacy of the healing agent. Different methods for assessing healing performance have been documented in the literature and direct comparisons are quite difficult to achieve. Nevertheless, the establishment of a standardized approach remains pending [38].

## 4. Conclusions

This work demonstrated the successful preparation of PA6/COC compatibilized blends to be potentially used as a self-healing matrix for thermoplastic structural composites. A detailed rheological, thermal, and mechanical characterization highlighted the positive contribution of the three different compatibilizers in tuning the repair capability of the prepared blends. From the analysis of the rheological behavior, in particular, from the trends of the storage modulus and complex viscosity, it was possible to understand the compatibilization effect played by E-GMA. FTIR analysis, together with the literature analysis, confirmed the production of a copolymer at the interface that lowered the surface tension of PA6 and thus enabled a better COC distribution for all three compatibilizers, especially with E-GMA. Consequently, the compatibilizers improved the toughness of the prepared blends in quasi-static mode, while, in impact mode, the Kic was similar to that detected in the uncompatibilized blend. These differences could be explained by analyzing the fracture surface. Upon the introduction of the three compatibilizers, the fracture surface in quasi-static mode appeared severely plasticized, thus promoting an increase in the toughness. On the other hand, in impact conditions, the compatibilizers did not substantially change the fracture morphology. The addition of the compatibilizers enhanced the healing efficiency of the prepared blends. In quasi-static mode, the healing efficiency of the uncompatibilized blend was 11.4%, while the addition of PE-g-MAH led to a healing efficiency of 13.7% (+20.2%), POE-g-MAH to 20.3% (+78.1%), and E-GMA up to 28.5%. (+150%). The situation dramatically changed in impact mode, where the compatibilizers played a much more pronounced effect. The healing efficiency of the uncompatibilized blend was 34.7%, PE-g-MAH led to a mild improvement in HE to 45.0% (+20.3%), POE-g-MAH led to a healing efficiency of 33.0% (−11.8%), while E-GMA enhanced HE up to 67.7% (+81.1%). The severe plasticization detected in quasi-static mode and introduced by the use of the compatibilizers hindered the optimal flow of the healing agents within the crack zone. On the other hand, in impact conditions, the fracture surface was more planar, and therefore the healing agent was capable of flowing undisturbed, thus properly filling the crack plane and leading to a higher repair capability. Overall, this work demonstrated the potential of PE-g-MAH, POE-g-MAH, and E-GMA in tuning the thermomechanical properties of PA6/COC blends. From this analysis, E-GMA resulted in being the best compatibilizer in terms of self-healing efficacy. Further studies will be performed in the future to deepen the knowledge of the role played by E-GMA compatibilizers in the PA6/COC system.

## Figures and Tables

**Table 1:** List of the prepared samples with the relative amount of constituents.

Sample	PA6 Content [wt%]	COC Content [wt%]	Compatibilizer Content [wt%]
PA6COC	70	30	0.0
5 wt% PE-g-MAH	66.5	28.5	5.0
5 wt% POE-g-MAH	66.5	28.5	5.0
5 wt% E-GMA	66.5	28.5	5.0

**Table 2:** Results of DSC tests on the prepared blends.

First Heating Scan					
Sample	T_g_ COC [°C]	T_m1_ PA6 [°C]	T_m2_ PA6 [°C]	ΔH_m_PA6_ [J/g]	χ PA6 [%]
PA6COC	64.0	221.5	224.7	36.6	22.7
5 wt% PE-g-MAH	62.8	216.2	224.5	42.0	27.5
5 wt% POE-g-MAH	63.0	217.8	224.2	41.9	27.4
5 wt% E-GMA	63.0	218.1	225.3	41.0	26.8
**Cooling scan**					
**Sample**	**T_c_ PA6** **[°C]**	**ΔH_c_PA6_** **[J/g]**	**χ PA6** **[%]**		
PA6COC	189.4	45.5	28.3		
5 wt% PE-g-MAH	190.8	47.6	31.4		
5 wt% POE-g-MAH	190.2	49.2	32.2		
5 wt% E-GMA	189.6	48.0	31.4		
**Second heating scan**					
**Sample**	**T_g_ COC [°C]**	**T_m1_ PA6** **[°C]**	**T_m2_ PA6** **[°C]**	**ΔH_m_PA6_** **[J/g]**	**χ PA6** **[%]**
PA6COC	61.0	219.3	224.0	45.6	28.3
5 wt% PE-g-MAH	62.3	217.5	224.3	42.9	28.0
5 wt% POE-g-MAH	62.3	217.3	224.3	43.1	28.2
5 wt% E-GMA	63.9	219.5	225.6	50.1	32.8

## Data Availability

Data are available upon reasonable request from the first author due to privacy.

## Supplementary Materials

The following supporting information can be downloaded at: https://www.mdpi.com/article/10.3390/ma17081880/s1, Figure S1: Optical microscope micrographs of the cross-section of the prepared blends.
