# Supplementary material for: Compatibilization of Polyamide 6/Cyclic Olefinic Copolymer Blends for the Development of Multifunctional Thermoplastic Composites with Self-Healing Capability
Source: Materials (Basel). 2024 Apr 18;17(8):1880. doi: 10.3390/ma17081880 (PMC11052209; doi:10.3390/ma17081880)
Supplement: Supplementary file 1 [file materials-17-01880-s001.zip › materials-2962910-supplementary.pdf]

Supplementary Material

Light microscope (LM) micrographs of the produced blends were acquired by embedding the specimens in cylindrical epoxy beads, polished, and subsequently analyzed with a CH-9435 Heerbrugg optical microscope (Heerbrugg). ImageJ® software (National Institutes of Health Campus, release 1.8) was used to measure the diameter of the COC domains inside the PA6 matrix. Figure S1 reports the micrographs used to determine the diameter of the COC domains.

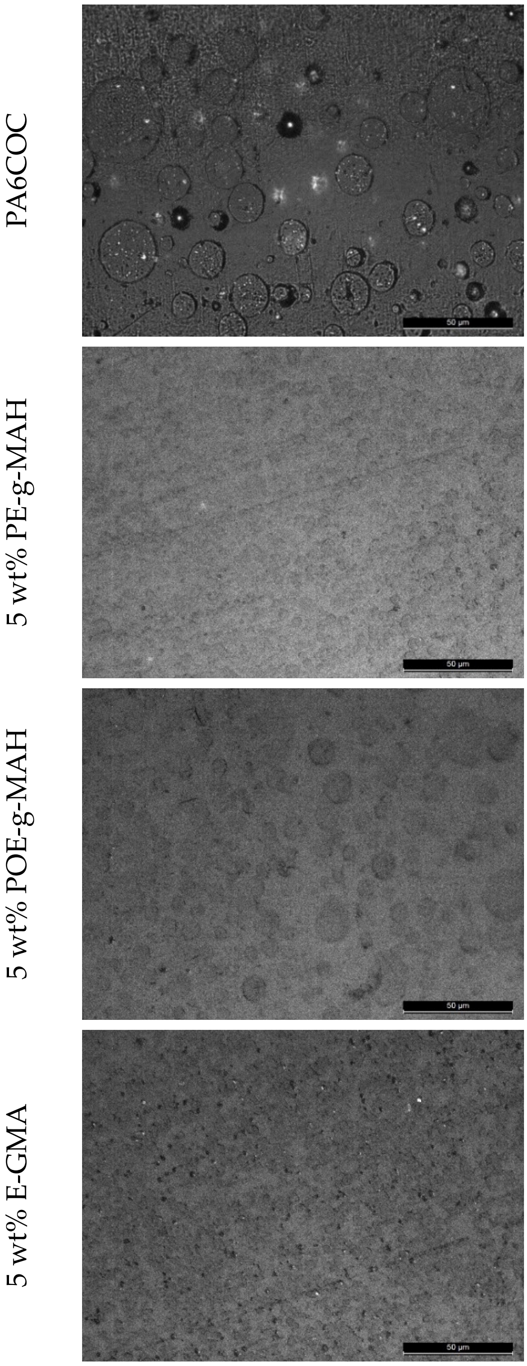

Figure S1: Optical microscope micrographs of the cross-section of the prepared blends.
